# Prolonged Neurological Depression, Bradycardia, and Gastroparesis Following Acute Oral Cannabinoid Ingestion: A Case Report

**DOI:** 10.7759/cureus.109647

**Published:** 2026-05-25

**Authors:** Nikos Adamidis, Sofia Adamidi, Vasiliki E Georgakopoulou, Athanasios Desalermos, Argyroula Karampela, Sofia Miliou, Eleni Valavani, Sotirios Adamidis

**Affiliations:** 1 First Department of Internal Medicine, Sismanogleio General Hospital, Athens, GRC; 2 Department of Internal Medicine, Charlton Memorial Hospital, Fall River, USA; 3 Department of Pathophysiology, Laiko General Hospital, Athens, GRC; 4 Medical School, Harvard University, Cambridge, USA; 5 First Department of Internal Medicine, Athens Medical Center, Athens, GRC; 6 Department of Primary Education, National and Kapodistrian University of Athens, Athens, GRC

**Keywords:** cannabinoid intoxication, cannabis edible, gastroparesis, sinus bradycardia, stroke mimic

## Abstract

Cannabis-containing edibles may be associated with clinically relevant intoxication, particularly when symptom onset is delayed and the clinical presentation is nonspecific. A 54-year-old man with treated dyslipidemia presented with worsening vertigo, dizziness, headache, slurred speech, psychomotor slowing, and generalized weakness after ingesting a cannabis-containing cookie. Symptoms began approximately three hours after ingestion and progressed to slurred speech and bradypsychia by 12 hours. On admission, he was awake but markedly bradykinetic and bradypsychic, with diffuse weakness and epigastric distension. Stroke workup, including brain CT, computed tomography angiography, and brain MRI, excluded intracranial hemorrhage, acute ischemia, and large-vessel occlusion. Chest CT was unremarkable, while abdominal CT showed marked gastric distension with retained gastric contents, compatible with acute gastric dysmotility/suspected gastroparesis, requiring Levin tube decompression. Cerebrospinal fluid studies and infectious testing were negative. Cardiac evaluation showed sinus bradycardia, with Holter monitoring revealing a mean heart rate of 48 beats/min and a nadir of 35 beats/min, without significant pauses or conduction abnormalities. Electroencephalography showed generalized slowing without epileptiform activity. Following consultation with the Poison Control Center, cannabinoid intoxication was considered the most likely diagnosis. The patient received supportive care with monitoring, intravenous fluids, and gastric decompression. His clinical condition improved from day three, and he was discharged fully recovered on day six. Cannabis-containing edible ingestion should be considered in the differential diagnosis of acute or subacute neurological symptoms when compatible exposure is reported. In this case, the presentation included bradypsychia, bradykinesia, slurred speech, bradycardia, and acute gastric dysmotility/suspected gastroparesis, initially raising concern for stroke or central nervous system infection. Careful diagnostic evaluation remains necessary to exclude serious alternative causes, but recognition of this possibility may help guide appropriate supportive management.

## Introduction

Cannabis-containing edible products have become increasingly available for both recreational and medicinal use and are often perceived as relatively safe, particularly because they do not involve inhalational exposure. However, edible cannabis preparations differ substantially from smoked or vaporized cannabis in their pharmacokinetic profile. Following oral ingestion, cannabinoids undergo gastrointestinal absorption and first-pass hepatic metabolism, resulting in delayed symptom onset, slower peak effects, and a more prolonged duration of action [1-3]. This delay may lead users to underestimate the potency of the product or consume additional quantities before the full effect has developed, increasing the risk of clinically significant intoxication.

The composition of edible cannabis products may also be unpredictable, especially when they are homemade or obtained from non-regulated sources. The concentration of Δ9-tetrahydrocannabinol (THC), cannabidiol (CBD), or other cannabinoids may be uncertain, and the clinical response may vary according to dose, individual susceptibility, co-ingested substances, comorbidities, and concomitant medications [1,3]. As a result, presentations related to cannabis edibles may be delayed, prolonged, and diagnostically challenging. THC is the principal psychoactive cannabinoid and acts mainly through cannabinoid receptor type 1 (CB1), which is widely expressed in the CNS, autonomic pathways, and enteric nervous system, whereas CBD has different pharmacological properties and does not produce the same classic psychoactive effects [1-3].

Acute cannabinoid intoxication can affect multiple organ systems. Neurological manifestations are among the most common and include dizziness, somnolence, ataxia, dysarthria, psychomotor slowing, confusion, and altered level of consciousness [4]. In some patients, these symptoms may resemble acute neurological emergencies, particularly posterior circulation stroke, seizure-related encephalopathy, metabolic encephalopathy, or CNS infection. This overlap is clinically important because symptoms such as vertigo, slurred speech, bradypsychia, and generalized weakness require urgent evaluation before intoxication can be accepted as the most likely explanation.

Cardiovascular manifestations may further complicate the clinical picture. Although tachycardia is the most frequently recognized acute cardiovascular effect of cannabis, bradycardia, hypotension, sinus pauses, and conduction abnormalities have also been reported, particularly after high-dose or oral cannabinoid exposure, and may reflect autonomic effects such as increased parasympathetic/vagal tone or reduced sympathetic activity [5,6]. Gastrointestinal effects are also biologically plausible, as cannabinoids may influence gastrointestinal motility through effects on the enteric nervous system. Delayed gastric emptying and gastroparesis-like manifestations have been described in relation to cannabinoid exposure, although they are less commonly emphasized in reports of acute intoxication [7].

We present a case of prolonged neurological depression, sinus bradycardia, and marked gastric distension compatible with gastroparesis after ingestion of a cannabis-containing cookie in a 54-year-old man. This case is clinically notable because it describes simultaneous neurological depression mimicking acute cerebrovascular disease, clinically relevant sinus bradycardia, and radiologically evident acute gastric dysmotility after edible cannabis ingestion in a middle-aged cannabis-naïve adult without major comorbidities.

## Case presentation

A 54-year-old man with a medical history of dyslipidemia, treated with rosuvastatin, presented to the ED with progressively worsening dizziness over approximately 10 hours, vertigo, and slurred speech after ingestion of a commercially obtained cannabis-containing cookie; however, the product was not available for toxicological or compositional testing, and the exact THC/CBD content, potency, dose, and cannabinoid composition remained unknown. According to the clinical history, symptoms began approximately three hours after ingestion, when he developed dizziness and headache severe enough to awaken him. Approximately 12 hours after ingestion, he developed slurred speech and marked psychomotor slowing. The patient reported no prior cannabis use, suggesting that he was cannabis-naïve. Reported symptom-onset intervals were based on the clinical history obtained at presentation and should therefore be interpreted as approximate patient-reported estimates.

On admission, vital signs were as follows: blood pressure 125/78 mmHg, heart rate 58 beats/min, respiratory rate 16 breaths/min, oxygen saturation 98% on room air, and temperature 36.7°C. The Glasgow Coma Scale score was 15/15. Neurological examination showed marked bradypsychia and bradykinesia but no focal neurological deficits. Cranial nerve examination was unremarkable, with no facial asymmetry, gaze deviation, dysphagia, or focal cranial nerve deficit. Motor strength was symmetric in all four limbs, graded 5/5 on the Medical Research Council scale, with no lateralizing weakness, sensory deficit, pathological reflex, or clear cerebellar sign. The NIH Stroke Scale/Score (NIHSS) score was 0, reflecting the absence of a focal stroke syndrome. He had persistent dizziness and abdominal distension, predominantly in the epigastric region.

Brain CT and computed tomography angiography of the brain and neck were negative for acute pathological findings, with no evidence of intracranial hemorrhage, acute ischemic changes, large-vessel occlusion, or posterior circulation vascular abnormality, including vertebrobasilar occlusion or significant stenosis (Figure 1).

**Figure 1:**
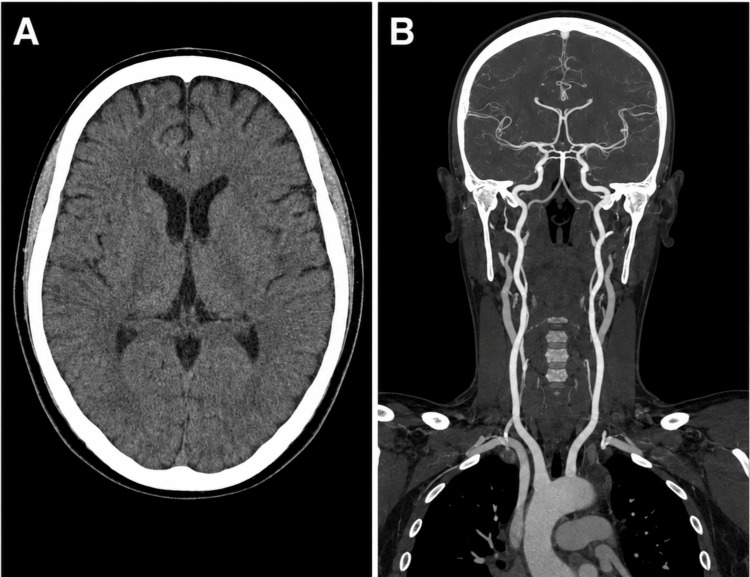
Brain CT and CT angiography of the head and neck. (A) Non-contrast brain CT showing no acute intracranial pathological findings. (B) CT angiography of the head and neck showing no large-vessel occlusion, significant arterial stenosis, aneurysm, or vascular malformation.

Brain MRI showed no evidence of intracranial hemorrhage, acute ischemic lesion, or occlusion of a major intracranial artery (Figure 2).

**Figure 2:**
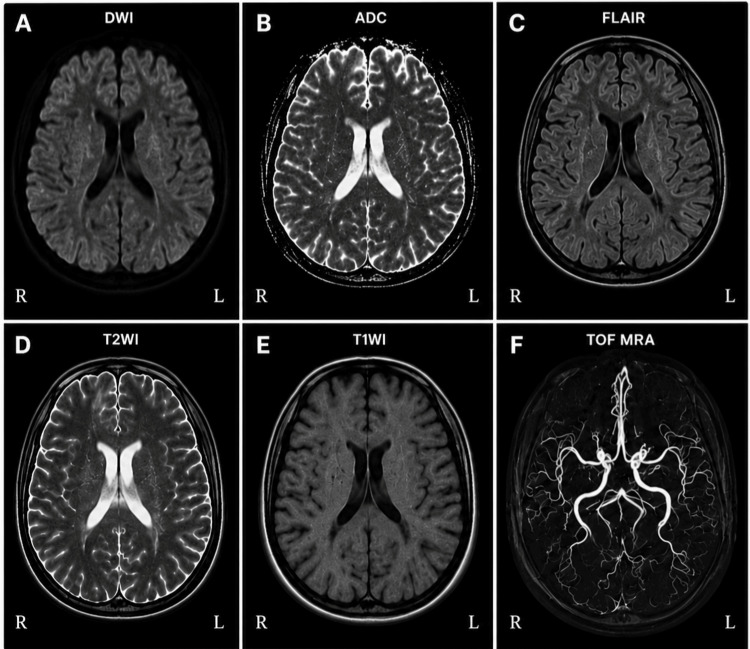
Brain MRI. (A) Axial diffusion-weighted imaging shows no areas of restricted diffusion. (B) Apparent diffusion coefficient map shows no corresponding low apparent diffusion coefficient signal. (C) Axial fluid-attenuated inversion recovery image shows no hyperintense lesion or edema. (D) Axial T2-weighted image shows no abnormal signal. (E) Axial T1-weighted image shows no mass lesion. (F) Time-of-flight magnetic resonance angiography shows preserved flow-related enhancement of the circle of Willis and major intracranial arteries, without evidence of major arterial occlusion, stenosis, or aneurysm. DWI: Diffusion-weighted imaging; ADC: Apparent diffusion coefficient; FLAIR: Fluid-attenuated inversion recovery; T2WI: T2-weighted imaging; T1WI: T1-weighted imaging; TOF MRA: Time-of-flight magnetic resonance angiography.

Chest CT was performed as part of the diagnostic work-up because of the patient’s prolonged depressed level of consciousness and concern for possible aspiration-related complications, as well as to further evaluate associated thoracoabdominal findings in the context of abdominal distension. Abdominal CT demonstrated marked gastric distension with retained gastric content, compatible with acute gastric dysmotility/suspected gastroparesis (Figure 3).

**Figure 3:**
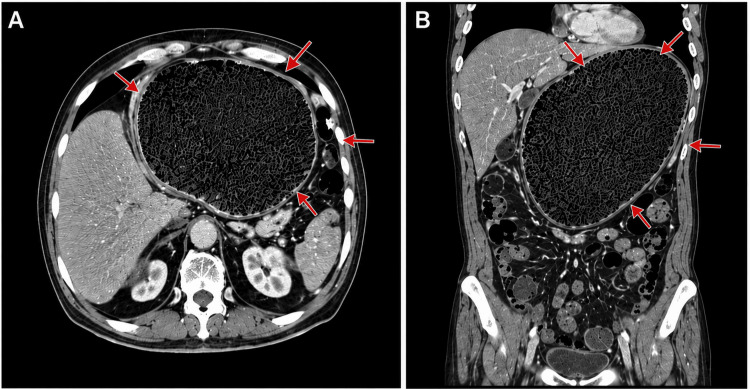
Abdominal CT showing gastric distension. Abdominal CT demonstrated marked gastric distension with retained gastric contents, compatible with acute gastric dysmotility/suspected gastroparesis in the appropriate clinical context. Red arrows indicate the markedly distended stomach. (A) Axial contrast-enhanced CT image of the upper abdomen showing a markedly distended stomach occupying most of the upper abdomen, with retained heterogeneous gastric contents and an air-fluid level. (B) Coronal contrast-enhanced CT reformatted image further demonstrating severe gastric distension extending to the mid-abdomen, without evidence of mechanical obstruction. These findings prompted gastric decompression with placement of a Levin nasogastric tube.

Surgical consultation was obtained, and a Levin nasogastric tube was inserted for gastric decompression, together with supportive IV fluid therapy.

The Poison Control Center noted that cannabinoid effects may be prolonged after oral ingestion. However, because the exact product composition and dose were unknown, no specific half-life could be reliably assigned in this case. Published pharmacokinetic data indicate that THC elimination is variable and multiphasic, with a prolonged terminal phase that may extend from approximately 1-3 days in occasional users and longer in chronic users [8]. This variability should be considered when interpreting the prolonged clinical course observed in this patient. Potential complications discussed included central nervous system depression, somnolence, lethargy, ataxia, respiratory depression, and cardiac arrhythmias, particularly in the setting of possible electrolyte disturbances. During hospitalization, however, no respiratory depression, oxygen desaturation, or abnormal respiratory rate was documented.

Further diagnostic workup was performed to exclude alternative neurological, infectious, and cardiological causes. No serum or urine cannabinoid quantification was available; therefore, biochemical confirmation of THC exposure or dose could not be obtained. This represents an important diagnostic limitation, and the diagnosis was based on the temporal association with ingestion of the cannabis-containing edible, the compatible clinical presentation, Poison Control Center consultation, and exclusion of alternative causes. Lumbar puncture yielded clear cerebrospinal fluid, with no evidence of pleocytosis, elevated protein, hypoglycorrhachia, or microbiological growth. Cerebrospinal fluid Gram stain, viral polymerase chain reaction testing, and culture were negative. Polymerase chain reaction testing of upper respiratory tract samples was also negative. The laboratory and microbiological findings are summarized in Table 1.

**Table 1:** Laboratory and microbiological findings from the diagnostic workup. CSF: Cerebrospinal fluid; LDH: Lactate dehydrogenase; μL: Microliter; mg/dL: Milligrams per deciliter; U/L: Units per liter.

Investigation/Sample	Finding	Unit of measurement	Reference range/expected result
Cerebrospinal fluid appearance	Clear	-	Clear
Cerebrospinal fluid Gram stain	Negative	-	Negative
Cerebrospinal fluid WBCs	3	cells/μL	0-5 cells/μL
Cerebrospinal fluid RBCs	10	cells/μL	0 cells/μL/none
Cerebrospinal fluid protein	35	mg/dL	15-45 mg/dL
Cerebrospinal fluid glucose	59.2	mg/dL	40-70 mg/dL
Cerebrospinal fluid lactate dehydrogenase	<15	U/L	<40 U/L
Cerebrospinal fluid viral PCR	Negative	-	Negative
Cerebrospinal fluid culture	Negative	-	Negative
Upper respiratory tract PCR	Negative	-	Negative
Serum potassium	4.1	mmol/L	3.5-5.1 mmol/L
Serum magnesium	1.9	mg/dL	1.7-2.4 mg/dL
Serum calcium	9.2	mg/dL	8.6-10.2 mg/dL
Thyroid-stimulating hormone	2.6	μIU/mL	0.4-4.0 μIU/mL

Cardiological evaluation included transthoracic echocardiography, which showed normal cardiac chamber dimensions, no significant valvular disease, and normal estimated pulmonary pressure (Figure 4).

**Figure 4:**
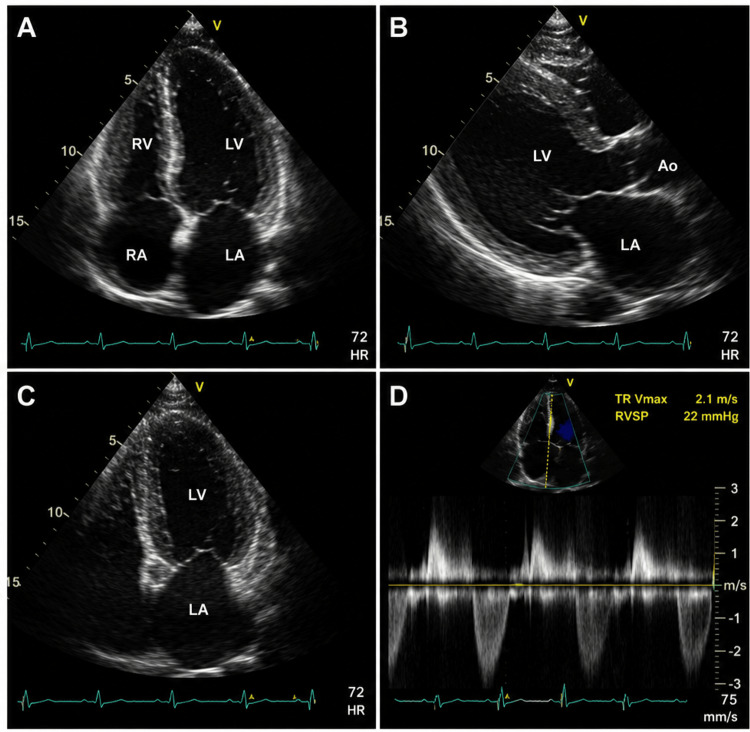
Transthoracic echocardiography. (A) Apical four-chamber view showing normal left and right ventricular size and systolic function, without pericardial effusion. (B) Parasternal long-axis view showing normal left ventricular size and wall motion, with normal aortic root and left atrial dimensions and no significant valvular abnormality. (C) Apical two-chamber view confirming preserved left ventricular systolic function. (D) Continuous-wave Doppler assessment of the tricuspid regurgitation jet showing an estimated right ventricular systolic pressure of 22 mmHg, consistent with normal estimated pulmonary pressure. Ao: Aorta; LA: Left atrium; LV: Left ventricle; RA: Right atrium; RV: Right ventricle; RVSP: Right ventricular systolic pressure; TR Vmax: Maximal tricuspid regurgitation velocity.

Holter monitoring demonstrated sinus bradycardia, with a mean heart rate of approximately 48 beats/min and a minimum heart rate of 35 beats/min. No pauses longer than 2.5 seconds were recorded, and there was no evidence of atrioventricular or intraventricular conduction disturbance. Reversible causes of bradycardia were also evaluated: serum potassium, magnesium, and calcium levels were assessed, thyroid dysfunction was excluded, and there was no use of beta-blockers, calcium-channel blockers, digoxin, antiarrhythmic agents, or other bradycardia-inducing medications.

Electroencephalography showed generalized slow activity, approximately 5-6 cycles per second, mainly within the theta range, intermittently interrupted by low-voltage beta activity, without definite epileptiform discharges (Figure 5). These findings were compatible with diffuse cerebral dysfunction, consistent with toxic-metabolic encephalopathy in the clinical context, and did not support an epileptic etiology.

**Figure 5:**
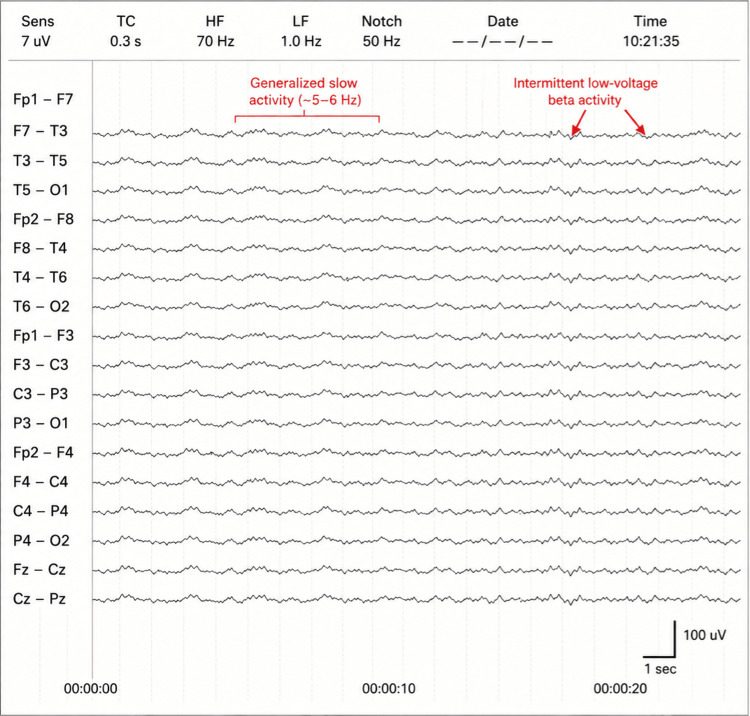
Electroencephalography during neurological assessment. Electroencephalography showed generalized slow background activity in the theta/delta range, without definite epileptiform discharges. Generalized slowing is a nonspecific marker of diffuse cerebral dysfunction and, in the present clinical context, was compatible with toxic-metabolic encephalopathy. The absence of epileptiform discharges made ongoing nonconvulsive seizure activity less likely. HF: High-frequency filter; LF: Low-frequency filter; TC: Time constant; μV: Microvolt; sec: Second.

During the first two hospital days, the patient remained bradykinetic and bradypsychic, with persistent slurred speech, while receiving nasogastric decompression and IV fluids. From the third hospital day, his neurological status progressively improved, and the Levin tube was removed. Oral intake was gradually reintroduced, and mobilization was initiated. By the fifth hospital day, he tolerated full oral feeding, and bradycardia had also improved. He was discharged on the sixth hospital day in a fully improved clinical condition.

Therapeutic intervention

The patient was managed conservatively and supportively. Treatment included close neurological and cardiorespiratory monitoring, IV fluids, and gastric decompression with a Levin nasogastric tube because of marked gastric distension with retained gastric contents, compatible with acute gastric dysmotility/suspected gastroparesis. No specific antidote was administered. The management strategy was guided by consultation with the Poison Control Center and serial clinical reassessment.

Outcome and follow-up

The patient showed gradual clinical improvement beginning on the third hospital day. Neurological manifestations, including bradypsychia, bradykinesia, and slurred speech, progressively resolved. Gastric decompression was discontinued after clinical improvement, and oral feeding was gradually resumed. Bradycardia improved during hospitalization. The patient was discharged on the sixth hospital day in a fully improved clinical condition.

Patient consent

Written informed consent was obtained from the patient for publication of this case report and any accompanying clinical images. All identifying information has been removed to protect patient confidentiality.

## Discussion

This case illustrates a prolonged multisystem toxicity syndrome following ingestion of a cannabis-containing edible, presenting with neurological depression, bradypsychia, bradykinesia, slurred speech, sinus bradycardia, and marked gastric distension with retained gastric contents, compatible with acute gastric dysmotility/suspected gastroparesis. Although acute cannabis intoxication is increasingly encountered in emergency settings, the combination of a stroke-like neurological presentation, clinically relevant bradycardia, and radiologically evident gastric dysmotility is uncommon and makes this case clinically notable.

The temporal pattern supports edible cannabinoid intoxication as the most plausible diagnosis. In contrast to inhaled cannabis, orally ingested cannabinoids are absorbed more slowly and undergo first-pass hepatic metabolism, leading to delayed onset, delayed peak effect, and prolonged duration of symptoms [1-3]. This pharmacokinetic profile explains why the patient initially developed dizziness and headache several hours after ingestion, followed by more prominent neurological manifestations approximately 12 hours later. The persistence of bradypsychia, bradykinesia, and slurred speech for two days, with gradual recovery over six days, is also consistent with prolonged oral cannabinoid effects rather than a rapidly resolving transient neurological event.

The initial clinical concern was acute cerebrovascular disease, especially because the patient presented with vertigo, slurred speech, psychomotor slowing, and generalized weakness. This diagnostic overlap has been highlighted by Finch and Vilke, who reported a woman presenting with acute extremity weakness and altered speech after accidental THC edible ingestion, triggering a stroke-code evaluation and raising concern for potential thrombolysis in a stroke mimic [9]. Our case is comparable in that the neurological presentation also prompted an acute stroke protocol. However, it differs in several important respects: our patient had more global psychomotor depression rather than focal hemiparesis, a more prolonged course, associated sinus bradycardia, and marked gastric distension requiring nasogastric decompression. Thus, our case extends the stroke-mimic phenotype of edible cannabis intoxication beyond focal motor symptoms to include prolonged encephalopathic and autonomic features.

Cardiovascular involvement was another important aspect of the presentation. Cannabis is more commonly associated with tachycardia, particularly after inhalational exposure or lower doses, but bradycardia has also been described after oral or high-dose cannabinoid exposure [5,6]. Ploucher S et al. reported a near-fatal case of accidental cannabis intoxication in an elderly man who presented with dizziness and altered mental status and developed severe bradycardia requiring atropine, with no alternative cardiac etiology identified after extensive evaluation [6]. Compared with that case, our patient was younger and did not require atropine or advanced cardiac intervention, but Holter monitoring documented clinically relevant sinus bradycardia, with a mean heart rate of 48 beats/min and a nadir of 35 beats/min. The absence of significant pauses, atrioventricular block, intraventricular conduction disturbance, or structural cardiac disease supported transient cannabinoid-related autonomic dysregulation rather than primary conduction system disease.

The rarity but clinical importance of bradycardia in cannabis toxicity is supported by poison-center data. Hendrickson RG et al. showed that heart-rate responses after acute cannabis exposure are variable and that bradycardia, although less common than tachycardia, may occur in clinically significant cannabis toxicity [10]. This observation is relevant to our case because the presence of bradycardia might otherwise have suggested a primary cardiac disorder, increased intracranial pressure, or another toxic-metabolic process.

In the appropriate clinical context, bradycardia should not exclude cannabis intoxication. Although tachycardia is the more commonly recognized cardiovascular response to cannabis, particularly at lower doses or after inhalational exposure, bradycardia has also been described, especially after higher-dose or oral cannabinoid exposure. This paradoxical response may reflect dose-dependent activation of the endocannabinoid-cardiac autonomic axis. Experimental and clinical data suggest that cannabinoid receptor type 1 (CB1) signaling can modulate autonomic cardiovascular control, including inhibition of sympathetic neurotransmission and enhancement of parasympathetic/vagal influence on heart rate [11]. Increased vagal tone may lead to sinus bradycardia, hypotension, sinus pauses, or, rarely, more severe conduction disturbances. Therefore, in this case, the documented sinus bradycardia, in the absence of structural heart disease, significant electrolyte abnormalities, thyroid dysfunction, conduction block, or bradycardia-inducing medication, was interpreted as compatible with transient cannabinoid-related autonomic dysregulation rather than primary cardiac conduction disease.

The gastrointestinal findings further distinguish the present case from many previously published reports. Abdominal CT demonstrated marked gastric distension with retained gastric contents, without evidence of mechanical obstruction. This was interpreted as acute gastric dysmotility/suspected gastroparesis in the appropriate clinical context, and the patient required gastric decompression with a Levin nasogastric tube. Cannabinoids can modulate gastrointestinal motility through CB1-mediated effects on the enteric nervous system, and delayed gastric emptying is biologically plausible in cannabinoid exposure [7]. The patient had no known history of gastrointestinal motility disorder and no prior recurrent gastrointestinal symptoms suggestive of baseline dysmotility. This presentation should also be distinguished from cannabinoid hyperemesis syndrome, which typically occurs in the setting of chronic cannabis use and is characterized by recurrent or cyclical nausea and vomiting, often associated with compulsive hot bathing behavior. In contrast, our patient reported no prior cannabis use, developed symptoms acutely after a single edible ingestion, and had no history of cyclical vomiting or previous similar episodes, making cannabinoid hyperemesis syndrome unlikely.

Cammarano CA and Villaluz JE reported a case of marijuana-induced gastroparesis with implications for aspiration risk in a patient considered nil per os, emphasizing that cannabis use may be associated with clinically meaningful delayed gastric emptying [12]. Our case differs because the gastric dysmotility occurred in the setting of acute edible intoxication with simultaneous neurological depression and bradycardia. This combination increases clinical risk because impaired consciousness and gastric retention may together predispose to aspiration, justifying close monitoring and early gastric decompression when significant distension is present. Alternative causes of gastroparesis were considered. Basic metabolic evaluation did not reveal clinically relevant electrolyte or metabolic abnormalities, and thyroid dysfunction was excluded. The patient had no known history of diabetes mellitus, prior delayed gastric emptying, recurrent postprandial fullness, chronic nausea or vomiting, or previous diagnosis of gastroparesis. He was not receiving medications commonly associated with impaired gastric motility. HbA1c was not available during admission, which represents a limitation. In this context, the acute onset after edible cannabis ingestion and improvement with supportive care favored transient cannabinoid-associated gastric dysmotility rather than established diabetic, post-viral, medication-induced, or chronic idiopathic gastroparesis.

The negative diagnostic workup was essential before attributing the syndrome to cannabinoid intoxication. Toxicology screening for other sedative or recreational substances was not available during admission, representing an additional diagnostic limitation; therefore, co-exposure could not be fully excluded. Nevertheless, neuroimaging excluded intracranial hemorrhage, acute ischemic lesion, and large-vessel occlusion; cerebrospinal fluid findings were non-inflammatory; and Gram stain, viral PCR, and culture were negative, making central nervous system infection unlikely.

Electroencephalography showed generalized slowing without epileptiform discharges, supporting diffuse cerebral dysfunction rather than nonconvulsive seizure activity. This pattern is nonspecific and should not be considered diagnostic of cannabinoid intoxication alone. However, previous studies of acute cannabis/THC exposure have reported changes in EEG background activity, including alterations in theta-band activity, while cannabinoid-related neurological case reports have variably described nonspecific slowing or epileptiform abnormalities depending on the clinical presentation. In the present case, the generalized slowing, absence of epileptiform discharges, compatible clinical context, negative neuroimaging, and gradual recovery supported a toxic-metabolic encephalopathic process. Cardiac evaluation excluded structural heart disease and high-risk conduction abnormalities. Therefore, the diagnosis was supported not by a single confirmatory test but by the combination of temporal exposure, compatible pharmacology, multisystem manifestations, exclusion of major alternative diagnoses, and spontaneous improvement with supportive care.

The exact cannabinoid composition of the ingested edible was not known in this case. Therefore, the concentrations of THC, CBD, or other cannabinoids could not be determined. This uncertainty should be considered when interpreting the clinical course, as the manifestations of cannabis-containing edibles may vary according to the ingested dose, cannabinoid profile, individual susceptibility, co-ingested substances, comorbidities, and concomitant medications. Accordingly, the prolonged neurological depression, bradycardia, and gastric dysmotility/suspected gastroparesis observed in this patient cannot be attributed to a specific cannabinoid concentration with certainty.

Compared with previously reported cases, the present case has several distinctive features. First, it involved a middle-aged adult with limited comorbidity and only dyslipidemia treated with rosuvastatin, whereas severe edible intoxication is often emphasized in pediatric or older adult populations [13,14]. Second, the presentation mimicked acute stroke but did not involve a clearly focal neurological deficit, broadening the spectrum of cannabis-related stroke mimics beyond the focal weakness and altered speech described by Finch and Vilke [9]. This interpretation was further supported by the absence of focal neurological deficits on examination.

Third, the patient had clinically documented bradycardia but no life-threatening pauses or conduction block, showing that significant sinus bradycardia may occur even without the need for pharmacologic chronotropic support, in contrast to more severe published cases requiring atropine [6]. Finally, the concurrent gastroparesis-like gastric distension requiring nasogastric decompression adds a gastrointestinal dimension that is less frequently reported in the acute edible intoxication case literature and links the neurological and autonomic toxicity of cannabinoids with clinically relevant gastrointestinal dysmotility [7,12].

Management of acute edible cannabinoid intoxication remains primarily supportive. In this case, close neurological and cardiorespiratory monitoring, intravenous fluids, exclusion of serious neurological and infectious diagnoses, Poison Control Center consultation, and nasogastric decompression were sufficient. No antidote was administered. The patient’s gradual improvement beginning on the third hospital day and complete clinical recovery by the sixth day further support the self-limited but potentially prolonged nature of oral cannabinoid toxicity. On follow-up after discharge, the patient remained clinically well, with complete resolution of neurological symptoms and no recurrent dizziness, slurred speech, bradypsychia, bradykinesia, abdominal distension, or other gastrointestinal symptoms. No recurrent cardiovascular symptoms were reported.

Concomitant medication use was also considered. The patient’s only chronic medication was rosuvastatin. A clinically significant pharmacokinetic interaction between rosuvastatin and the ingested cannabinoid product was considered unlikely. Rosuvastatin undergoes limited CYP-mediated metabolism and is not primarily metabolized by CYP3A4; its disposition is more dependent on hepatic uptake/efflux transporters, including OATP1B1 and BCRP. In contrast, THC and CBD are metabolized mainly through hepatic CYP pathways, including CYP2C9, CYP2C19, and CYP3A4 [15-17]. Therefore, although cannabinoid-drug interactions should be considered in cases of intoxication, rosuvastatin was not considered a likely contributor to the prolonged neurological depression, bradycardia, or gastroparesis observed in this case.

This case reinforces several practical messages. Clinicians should actively ask about edible cannabis exposure when evaluating unexplained altered mental status, dysarthria, vertigo, bradypsychia, bradycardia, or acute gastric distension. A history of edible ingestion should not prematurely terminate the evaluation of stroke, encephalitis, seizure, or cardiac conduction disease, but it should be integrated early into the differential diagnosis. Finally, hospitalization and prolonged observation may be required when edible intoxication is accompanied by neurological depression, clinically significant bradycardia, or gastrointestinal dysmotility.

## Conclusions

This case describes a prolonged multisystem presentation temporally associated with ingestion of a cannabis-containing edible, characterized by neurological depression, stroke-like symptoms, sinus bradycardia, and marked gastric distension compatible with acute gastric dysmotility/suspected gastroparesis. Although confirmatory cannabinoid quantification was not available, the clinical course, exclusion of major alternative neurological, infectious, metabolic, and cardiac causes, and gradual recovery with supportive care were consistent with edible cannabinoid intoxication. This case suggests that edible cannabis exposure should be considered in the differential diagnosis of unexplained neurological depression or stroke-mimic presentations, particularly when accompanied by cardiovascular or gastrointestinal findings. In such cases, clinicians should obtain a careful exposure history, including the route of cannabis use and possible edible ingestion, while continuing to exclude time-sensitive alternative diagnoses such as stroke, central nervous system infection, seizure-related encephalopathy, metabolic disturbance, and primary cardiac rhythm disorder.

## References

[1] Barrus DG, Capogrossi KL, Cates SC (2016). Tasty THC: promises and challenges of cannabis edibles. Methods Rep RTI Press.

[2] Grotenhermen F (2003). Pharmacokinetics and pharmacodynamics of cannabinoids. Clin Pharmacokinet.

[3] Huestis MA (2007). Human cannabinoid pharmacokinetics. Chem Biodivers.

[4] Baraniecki R, Panchal P, Malhotra DD, Aliferis A, Zia Z (2021). Acute cannabis intoxication in the emergency department: the effect of legalization. BMC Emerg Med.

[5] Goyal H, Awad HH, Ghali JK (2017). Role of cannabis in cardiovascular disorders. J Thorac Dis.

[6] Ploucher S, Koilpillai S, Iyyani M, Carlan S (2023). A case of near-fatal bradycardia caused by accidental cannabis intoxication. Cureus.

[7] Camilleri M (2018). Cannabinoids and gastrointestinal motility: pharmacology, clinical effects, and potential therapeutics in humans. Neurogastroenterol Motil.

[8] Chayasirisobhon S (2020). Mechanisms of action and pharmacokinetics of cannabis. Perm J.

[9] Finch NA, Vilke GM (2020). Unknown tetrahydrocannabinol edible ingestion resulting in acute stroke presentation. J Emerg Med.

[10] Hendrickson RG, Hughes AR, Kusin SG, Lopez AM (2021). Variation in heart rate after acute cannabis exposure. Toxicol Com.

[11] Heckle MR, Nayyar M, Sinclair SE, Weber KT (2018). Cannabinoids and symptomatic bradycardia. Am J Med Sci.

[12] Cammarano CA, Villaluz JE (2021). A reason to rethink fasting guidelines? Marijuana-induced gastroparesis and the implications for aspiration risk in the nil per os (NPO) patient: a case report. Am J Case Rep.

[13] Hendrickson RG, McKeown NJ, Kusin SG, Lopez AM (2020). Acute cannabis toxicity in older adults. Toxicol Comm.

[14] Idris I, Diez JR, Assoku BA, Beker S (2022). Accidental ingestion of tetrahydrocannabinol-laced gummies causing bradycardia and first-degree atrioventricular block in a pediatric patient: a case report. Cureus.

[15] Elsby R, Coghlan H, Edgerton J, Hodgson D, Outteridge S, Atkinson H (2023). Mechanistic in vitro studies indicate that the clinical drug-drug interactions between protease inhibitors and rosuvastatin are driven by inhibition of intestinal BCRP and hepatic OATP1B1 with minimal contribution from OATP1B3, NTCP and OAT3. Pharmacol Res Perspect.

[16] Yabut KC, Winnie Wen Y, Simon KT, Isoherranen N (2024). CYP2C9, CYP3A, and CYP2C19 metabolize Δ9-tetrahydrocannabinol to multiple metabolites, but metabolism is affected by human liver fatty acid-binding protein (FABP1). Biochem Pharmacol.

[17] Doohan PT, Oldfield LD, Arnold JC, Anderson LL (2021). Cannabinoid interactions with cytochrome P450 drug metabolism: a full-spectrum characterization. AAPS J.

